# “In Their Own Voice”—Incorporating Underlying Social Determinants into Aboriginal Health Promotion Programs

**DOI:** 10.3390/ijerph15071514

**Published:** 2018-07-18

**Authors:** Shannen Vallesi, Lisa Wood, Lyn Dimer, Michelle Zada

**Affiliations:** 1 School of Population and Global Health (M431), The University of Western Australia, Crawley, WA 6009, Australia; lisa.wood@uwa.edu.au; 2 Heart Foundation WA Division, Subiaco, WA 6008, Australia; lyn.dimer@heartfoundation.org.au (L.D.); michelle.zada@heartfoundation.org.au (M.Z.)

**Keywords:** Aboriginal and Torres Strait Islander, social determinants of health, cardiac rehabilitation, health promotion program, Indigenous, chronic disease management, cultural competence, cultural safety

## Abstract

Despite growing acknowledgement of the socially determined nature of health disparities among Aboriginal people, how to respond to this within health promotion programs can be challenging. The legacy of Australia’s assimilation policies have left profound consequences, including social marginalisation, limited educational opportunities, normalisation of premature death, and entrenched trauma. These social determinants, in conjunction with a reluctance to trust authorities, create barriers to accessing healthcare services for the prevention, treatment, and rehabilitation of chronic disease. The Heart Health program is a culturally sensitive cardiac rehabilitation program run at the local Aboriginal Medical Service in Perth, Western Australia that has since moved beyond cardiac education to provide a holistic approach to chronic disease management. A participatory action research framework was used to explore Heart Health participant and service provider perspectives on the barriers, enablers, and critical success factors to program participation and behaviour change. Thematic analysis of interview transcripts was undertaken, and through yarning (Aboriginal storytelling) sessions, many participants made unprompted reference to the impacts of white settlement, discrimination, and the forced fracturing of Aboriginal families, which have been explored in this paper reiterating the need for a social determinants lens to be taken when planning and implementing Aboriginal health promotion programs.

Aboriginal Health means not just the physical well-being of an individual but the social, emotional and cultural well-being of the whole Community in which each individual is able to achieve their full potential as a human being, thereby bringing about the total well-being of their Community.

National Aboriginal Health Strategy (1989) [1].

## 1. Introduction

Cardiovascular disease, diabetes, and other chronic lifestyle diseases are starkly over-represented in Aboriginal and Indigenous populations around the world, including Australia [2]. These preventable diseases have ravaged Aboriginal and Torres Strait Islander (Aboriginal) communities across Australia, contributing to the decade difference in life-expectancy between Aboriginal and non-Aboriginal Australians [3,4]. The prevailing inequalities in Aboriginal health results from a constellation of inter-related historical, cultural, political, psychosocial, and environmental factors [5,6], contributing to engagement (or lack thereof) in programs seeking to improve health outcomes [7,8].

As noted by Marmot (2011), inequalities in health arise from inequalities in society, with key determinants influenced by the circumstances in which people are born, grow, live, work, and age [5]. Whilst health promotion programs are traditionally framed around disease prevention and behavioural risk factors [9], inadequate consideration of the underlying social determinants of health can be a massive impediment to program participation and outcomes [10]. In the published literature, the work of Matthews [11] powerfully illustrates the impact of white colonisation on Aboriginal health and the need for health professionals to become better prepared to deliver interventions in a culturally appropriate manner. Underlying social determinants of health have been identified as barriers to health service engagement [12], compounded further in Aboriginal communities by the pervasive influence of inter-generational trauma [13]. Rasanathan et al. (2011) go so far as to argue that “health services that do not consciously address social determinants exacerbate health inequities” [12] (p. 656), and this is reflected in disparities of healthcare for Aboriginal people [14] and evidence of unfair treatment by the health system [3,15,16,17,18,19].

Health for Aboriginal people cannot be conceptualised as just one aspect of life, and reductionist interventions that focus exclusively on particular body parts or diseases often fail [20]. Empowering Aboriginal Australians to determine how health programs and preventive services can best support their health outcomes is an important endeavour in contributing to the closing the disparity gap with non-Aboriginal people. There is, however, a paucity of available programs designed and implemented by Aboriginal people themselves [7]. An exception is the Heart Health program, which unlike more mainstream health programs, has been intentionally designed and structured in a way that is culturally secure and tailored to Aboriginal people. With a strong ethos of flexibility and recognition of the array of social determinants of Aboriginal health and wellbeing [21], Heart Health differs substantially from traditional hospital cardiac rehabilitation programs (see Table 1).

Heart disease is a major preventable cause of premature death and disease among Aboriginal people, and this culturally secure program was developed with and for Aboriginal people in order to facilitate lifestyle and health changes to reduce cardiovascular disease. While there have been many reports about the health disparities experienced by Aboriginal people and programs designed to address these [16], there are many programs that have failed in reducing these gaps. In contrast, the Heart Health program stands out as a rare example of a program that has garnered active participant engagement, with many clients attending through “word of mouth”.

This study responds to compelling calls for more practice-driven evidence in health promotion [22], and for the co-creation of useful knowledge by researchers alongside practitioners and community participants [23]. The overarching aim of the project was to capture through the eyes and voices of Aboriginal people, the barriers, enablers, and critical success factors to program participation and behaviour change. This paper foremost explores the raft of underlying and contextual determinants of Aboriginal wellbeing and how these shape participation in the Heart Health program.

## 2. Materials and Methods

As Aboriginal people traditionally favour oral and visual communication [19], a participatory action research (PAR) methodology of photovoice was chosen as a culturally appropriate method to promote dialogue and knowledge sharing [24,25,26,27,28]. Photovoice provides individuals the opportunity to take photographs and facilitates discussion to explore participant experiences of the Heart Health program [29,30].

### 2.1. Study Setting

Interviews were conducted throughout 2016 at Derbarl Yerrigan Health Service (DYHS)—an Aboriginal Medical Service (AMS) which Heart Health operates out of every Thursday morning since 2009.

### 2.2. Participant Selection

A reference group was established comprising of seven participants, including Heart Health program participants (*n* = 2), staff representatives from DYHS and the Heart Health program (*n* = 3), and Heart Foundation representatives (*n* = 2) to help guide the research design and provide suggestions on key participants, staff, and stakeholders to engage. Reference group members were either key staff involved in program delivery or participants who had been involved in the program since its inception. Two of this paper’s authors were involved in the reference group and work at a local community health promotion organisation. The reference group only formally met prior to the study commencement, but was informally involved throughout the study to provide feedback and recommendations.

Initial recruitment began in March 2016 and involved displaying posters at DYHS, promoting the evaluation via a brief presentation during morning tea, and working with the reference group to identify individuals that may be interested in being involved.

Staff and stakeholders were approached to capture their observations regarding participants. These interviews provided alternative perceptions as to why clients come along, what the barriers and enablers are to program participation, and insights into the critical success factors of the Heart Health program that are required if the program were to be duplicated.

### 2.3. Data Collection

Photovoice was originally developed by Wang and Burris [30] as a way to engage those who are traditionally disenfranchised or without a voice, and has become recognised as an engaging, empowering, and culturally appropriate when researching with Aboriginal communities [31,32]. The visual element of photovoice also helps “break the ice” between the individual and the researcher [33] promoting dialogue and knowledge sharing through the discussion of photographs within a safe environment. Through photovoice, participants are involved in identifying and collecting “data” and then have the opportunity to share the stories behind their “data”, hence the method creates a sense of participant ownership of the research process and its outcomes [33]. Overall, the methodology differed slightly per participant availability and ability but involved three main components:

(1) An initial group or individual discussion where the study was explained, consent was obtained, information packs (including cameras, research questions, and instructions) were provided, and questions were answered;
(2) participants went away with their cameras and took photos loosely based on the three research questions; and
(3) photos were then used to facilitate the yarning (story telling) sessions with the authors to address the research questions.

Data were generated from in-depth, unstructured interviews conducted face-to-face with clients, staff, and stakeholders. Interviews were recorded and additional reflective notes recorded. No formal interview guide was established to enable participants to direct the conversation more organically, with the research team making it clear to participants that the interviews were about them and their photos, not about the research team asking them questions. However, the research team had three overarching questions for the evaluation:

(1) Why do you come to the Heart Health program (what motivated you to start coming, what motivates you to keep coming, what things make it hard for you to come along sometimes)?
(2) What changes have you made in your life since being involved in the Heart Health program (have you seen any benefits from the changes you have made)?
(3) Have you shared the information you have learnt from the Heart Health program with other people (e.g., friends or family)?

Overall, 26 Heart Health clients were approached or self-referred to participate in the evaluation; of these, 16 clients participated. While there were a number of individuals who indicated they were interested in participating, due to life circumstances at the time they were unable to participate. Additionally, 13 DYHS/Heart Health staff and stakeholders participated in semi-structured, qualitative interviews; no staff opted to participate in the photo taking aspect of the project. A total of 564 photographs were taken by participants.

### 2.4. Data Analysis

Interviews were transcribed verbatim by Pacific Transcription and coded using QSR NViVo following each interview. Data was extrapolated from participant’s narratives relating to photographs taken. Thematic analysis using inductive category development and contact comparison coding was undertaken with cross-checking between team members to enhance validity. The coding schema was not static and was revised to include emergent codes as data analysis progressed. To ensure that all conclusions in the study were dependent upon the subjects and not the researcher, key findings were presented to the authors for discussion.

Approval to conduct this research project was granted by the Western Australian Aboriginal Health Ethics Committee on 11 December 2015. Details are WAAHEC HREC Project Reference: 682, approved on 11 December 2015.

## 3. Results

In exploring the motivators, enablers, and barriers to Heart Health program participation, a foremost finding of the study is that these are substantially shaped by a raft of underlying and contextual determinants of Aboriginal wellbeing. This is by no means a new revelation to Aboriginal people, but the influence of these distal factors on health behaviours and health service engagement is often not adequately taken into account in programs ostensibly designed for Aboriginal people [34]. As articulated in a 2015 Canadian systematic review of social determinants of health influencing Indigenous health: “Efforts to overcome the ongoing health inequities amongst Indigenous peoples should involve understanding the contextual determinants of health and the interactions between these broader systemic environments and individuals’ responses to these circumstances [34].”

**Figure 1:**
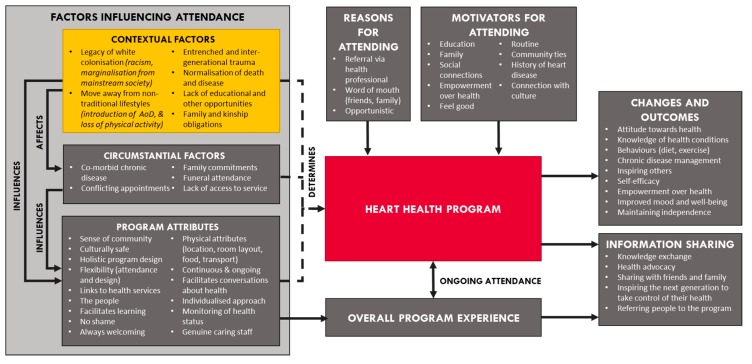
Factors influencing Heart Health program attendance and associated outcomes.

Figure 1 provides a visual synthesis of the key contextual and socially determined factors that emerged unprompted in the yarning and interviews with participants. It is important to note that these six themes are not an exhaustive list of all social determinants experienced by participants, but rather reflect the factors raised when participants were asked about their Heart Health journey. Whilst Figure 1 also depicts other proximal and program related factors that emerged as motivators, enablers, and barriers to participation, the focus of this paper is prima facie on the social determinants and these six themes discussed below.

### 3.1. Legacy of White Colonisation

The colonisation of Australia and the consequential assimilation policies and ensuing discrimination [35] has had many ripple effects on the health of Aboriginal people. The recent rejection of the Uluru Statement from the Heart (a statement delivered on behalf of Aboriginal and Torres Strait Islander people at the 2017 National Constitutional Convention calling for a constitutionally enshrined voice in parliament, as well as paving the way for a formal treaty between Aboriginal people and federal and state governments [36]) by the Australian Government further reiterates the lack of constitutional recognition of the legacy of our first peoples [37]. With the historical removal of children by white, Anglo-Saxon authorities, the consequences are still apparent many decades later, with participants noting their distrust of “white people” and people of authority throughout this evaluation. With other studies finding significant links between lack of trust and reluctance to engage in health programs [8].

Where I come from I wasn’t able to talk, to look at people, white people…I worked at community welfare for a while. When anyone comes to the desk you have to attend to them. I had great difficulty with that…If it was a policeman, I would not go near them.

What was found for the Heart Health program, was despite numerous Heart Health staff and weekly guest-presenters not being Aboriginal themselves, an underlying respect for participants and a holistic approach to healthcare provision nurtures a culture of safety. The Heart Health culture is one that is free of shame allowing participants opportunities to discuss their health experiences and journeys to Heart Health.

The fact that you talk to my Elders with respect in a caring manner I want to engage with you. So that’s the beauty of this program…it allows us to address our health culturally with our families and our aunties and our uncles and our Elders.

One way in which this is achieved, is through the “mini-workshops” on culturally appropriate communication styles that are provided to guest speakers prior to their presentations to the Heart Health group.

…if you’re going to get guest speakers in—so when the guys came from the universities… I meet them the week before and spend about an hour-and-a-half to two-hours teaching them how to yarn to a group, and how to present in a way that the clients would actually get it, rather than them trying to give a university format presentation.

### 3.2. Impact of Moving Away from Traditional Lifestyles

The colonisation of Australia brought with it exposure to alcohol and a major disruption to the traditional diet and hunter-gatherer lifestyles of Aboriginal people [38], and this has had a significant impact on health disparities [39]. Chronic diseases related to diet and lifestyle are over-presented in Aboriginal people. Aboriginal people are 3.3 times more likely to suffer from diabetes, are 1.2 times more likely to have cardiovascular disease, and 1.6 times more likely to be obese than non-Aboriginal Australians [3,40]. This disproportionate burden of disease and risk factors reflects how Aboriginal health has been shaped drastically by loss of identity, loss of family, racism, and trauma [41]. Comments arising from this evaluation discussed the impact “westernisation” has had on traditional lifestyles.

I’m a hunter, I’m a Noongar (original inhabitants of the geographical area in the south-west of Western Australia), and we used to go hunting a lot…so walking’s always been in my life and culture. It was not until the white man came that we got cars and we decided not to walk. So I’ve always walked. I’ve walked off the reserve into schools. I’ve walked off things, so walking’s always been part of my life.

The Heart Health program has responded to this in a variety of ways, including the incorporation of group physical exercise sessions, chronic disease education, diabetes and blood pressure monitoring, healthy meals, cooking sessions, and access and referral to primary care services.

Importantly, cultural appropriateness and relevance is not an “add-on” but a central tenet to the way in which program content is designed and delivered. For example, during the exercise sessions (run by an exercise physiologist), rather than instructing them to do “this stretch” or “that stretch”, exercises are tailored to be relevant to culture:

The other week, we did running like a dingo… and [imagine] walking through the bush…

Valuing and recognition of culture is embedded in other ways throughout the year, for example, the inclusion of bush tucker (traditional Aboriginal food) cooking demonstrations and planning of activities around NAIDOC week (a yearly Australian celebration of Aboriginal achievements and culture) [36].

There is also a strong emphasis on the role of the program in facilitating social connection, and additional time is built into the program structure to enable people to interact informally before, during, and after the program.

Communication is part of the thing that keeps people together. When Aboriginal people lived on reserves they were a group of people that communicated, they helped each other, they talked to people. You talk about a village raises a child, this is exactly what happened on reserves, years ago, when people actually looked after each other. You took concern for each other, you talked to each other. Somebody’s kid did something wrong, you told that kid off. They said, oh, thank you for stopping my kid from doing something silly. The white man come along and isolated everybody.

But, no, but seriously in a holistic approach you fellows want us to address our diabetes and just talk about your toes drop off and things like that. Well things drop off and we shouldn’t not talk about it…

The social interaction and connectedness fostered within the program has been observed to have reciprocal health promotion benefits, with participants often discussing their health, sharing healthy eating ideas, or cajoling one another to join in with the exercises or the weekly walk around the block.

We all talk about our [illness]. So that’s good because I’ve never seen that anywhere else where you are sitting down talking about what your numbers are for your diabetes and all that.

### 3.3. Hindrance of Educational Opportunities

Aboriginal Australians face numerous, well-documented barriers to accessing health. One theme that was raised on numerous occasions was the hindrance of educational opportunities (to themselves directly or to their parents) pertaining to both schooling and more specifically to health education. Education shapes individuals’ knowledge and skills that inform choices and behaviours that affect health, it also impacts opportunities for further education and employment and has implications for socio-economic status [42,43].

I was told I was dumb in primary school. I would never amount to anything, and that I had the lowest IQ. So I did give up a bit of the schooling, and concentrated on the sports.

…my pop taught me to write really early…I was his little secretary…When I got to high school, I loved it. For someone who was supposed to be that dumb at maths, all I wanted was to do long division, someone to show me and my teacher, she wouldn’t come to show me. That’s all I wanted.

The obstacles to their own education has made many of the participants passionate about education for the next generation:

All I wanted, was him [her son] to have a good education. So I think that’s one thing I wanted more than anything because I never had the chance. I was stopped.

A longing for health knowledge earlier in life to prevent lifestyle diseases from developing was also noted. Some participants reflected that they were grateful about all the learnings passed on from their parents, but also noted limitations regarding lack of health knowledge. As pragmatically noted by some participants, it is too late to prevent some of their chronic diseases/ailments, but this has fueled their passion for the next generation to learn more about the effects of sugar, diabetes and unhealthy eating.

I’d have loved to have learned it when I was 15 years old…But they [my parents] didn’t teach us about health because they didn’t know about health themselves.

In a paradoxical approach to conventional programs that are often of a fixed duration (e.g., five or six weeks), Heart Health has been intentionally designed with flexibility and inclusion in mind. Participants cycle in and out of the program freely; healthy food is provided; socialisation is encouraged; and education sessions are in multiple different formats (i.e., yarning circles, video, hands-on demonstrations) and not in a strict “you must attend this six-week course” style.

…learn to be flexible, and that’s why the diabetic education is one that you can actually—if you miss out in one lecture or you come in half way through, you just go back and start again. So it’s just a continuous circle, you know? And that’s how things are with Aboriginal education. Everything’s in circles, not in lines.

The cornerstone for the Heart Health program is Aboriginal empowerment, and to take responsibility over one’s own health. This underlying philosophy is fortified throughout all aspects of program delivery, as a result, multiple different mediums are used to help bolster learnings and the approach of “with, not for” people is taken. One example of how this is done, is making participants demonstrate how they administer their insulin injections.

It’s interesting watching some of the nursing students…because they’ll give it to the client and the client’s got no idea. Like [client] when he used to come in at the beginning and he had no idea what he was doing and we couldn’t work out why his sugars were ridiculous, but he used to pop it in, go boom and then take it straight out and it [the insulin] would be spraying all over the floor. So no wonder his sugars are 26 [mmol/L]. So then it’s like push your finger on it and count up to 10.

### 3.4. Entrenched and Inter-Generational Trauma

There is growing acknowledgment of the cumulative impacts of decades of inter-generational or transgenerational trauma on Aboriginal people in Australia [13] and in countries such as Canada [44] where similar colonisation and assimilation policies prevailed. Transgenerational trauma has been defined as “a collective complex trauma inflicted on a group of people who share a specific group identity or affiliation, ethnicity, nationality, or religious affiliation [and] the legacy of numerous traumatic events a community experiences over generations”[45] (p. 320).

Two of the five essential elements for responding to collective, generational Aboriginal trauma according to Krieg [46] is promoting a sense of safety and promoting connectedness, something fundamental to Heart Health’s model of care. Providing participants with a safe space to share their experiences is established through a number of mediums: continuity of staff, a room layout conducive to sharing, and always making people feel welcomed.

I try and create an atmosphere where it’s not intimidating. So if you haven’t done your sugars for three months or had your tablets for two weeks I’m not going to make you wrong for that…

Entrenched trauma has flow-on effects to mental health and health behaviours, and as such, one of the benefits of Heart Health being co-located at an AMS is the availability of an on-site trauma worker. This has reciprocal benefits to both the program and to DYHS, as many Aboriginal clients feel safe and have trust at Heart Health so would prefer to see someone at DYHS (rather than externally) for social and emotional wellbeing. Stolen Generation (a period of forced removal of Aboriginal children from their families by church missionaries and statutory welfare bodies between 1910 and 1970, as part of state and federal assimilation policies) camps are run as part of DYHS services, and are available to Heart Health clients to provide opportunities to grieve and heal on their country.

I came on board as the Stolen Gen worker…I come in here [Heart Health] to say hello to my Stolen Gen clients. That’s my time to give them information, see them about camps, see how they’re going, just check on generally how they’re feeling, if they want any help and support…

At the end of the day, everybody who walks in this building is a client of Derbarl. Everybody who walks in here is a potential client of Stolen Gen because at least 98 per cent of them are first, second or third generation. Or their kids or grandkids or their niece or nephew or someone has been taken away and removed, whether it’s the old Native Welfare, the DCP (Department of Child Protection (now the Department of Communities, Child Protection and Family Support)), whoever.

Adding to the pervasiveness of inter-generational trauma associated with colonisation and the Stolen Generation, the health and social disparities faced by Aboriginal people contribute further to cumulative trauma across the life-course. As reflected in the lives of the participants interviewed in the Heart Health program, Aboriginal people are more likely than other Australians to have family members die early or be incarcerated or engaged with the child protection system [47], and far more likely to face racial discrimination and stigma [48].

I was 15 years old when I lost my mum. She had terminal cancer…As children we had to deal with it and there was nothing out there as any counselling. There was nothing. We just had to deal with it the best we can. My dad hit a brick wall. He just drowned himself in alcohol…

But I was raised on a reserve and that’s where all of these things happened. Like the drinking, fighting, belting. I was only 13 or 14 I think.

The last decade has witnessed growing attention to the need for trauma informed practice in community and health services [49], and in the shadow of this is a growing discourse around trauma’s implication in drug, alcohol, and tobacco use [50,51].

I do alcohol and I do cigarettes…but when it’s instilled in a person—you know—and … looking at what you see your parents do, looking at what you see your uncles do and the whole family, living on a reserve...it just becomes the norm.

### 3.5. The Normalisation of Premature Death and Inevitability of Disease

Aboriginal people in Australia have a life expectancy 10 years younger than non-Aboriginal Australians, and are disproportionately represented across nearly all chronic disease and morbidity statistics, and efforts to close these gaps are making slow progress [52]. As a consequence, premature death and illness has become normalised in Aboriginal families reflected through comments by many participants.

I am 55 years old. My uncles, that is my dad’s brothers, I thought they were old and they were only 40 when they passed away.

Reiterating the necessity of earlier health education for individuals, participants reflected on how they always believed they were always going to have heart issues or get diabetes as it is a part of their family. The prevalence of these chronic health diseases is so engrained in their life course, that it almost appeared inevitable that this was going to be their own experience, therefore indulgence in risky lifestyle behaviours were irrelevant.

I looked at diabetes as a generational thing in my mob…my mum and my aunties…Generationally it hasn’t changed too much…I just thought it happened to people mainly women because that’s what I grew up with…sometimes there’s an expectation my family’s got it so I’m going to get it so it doesn’t matter.

My dad—he’s had a CABG [Coronary Artery Bypass Grafting] and a triple bypass…His sister’s had triple bypasses. His brother and my mother’s brother have had heart disease—my uncle—a heart attack…

As well as the heightened sense of fragile mortality that this can instil in individuals and communities, this also means that funerals and mourning are part of day-to-day life for many Aboriginal people [53,54]. The centrality of extended family in Aboriginal culture, and the kinship obligations that accompany this, means that funerals are sadly much more a part of “day-to-day” life for Aboriginal people in Australia [54]. Attending the funerals of family members is important, and this is overtly respected within the Heart Health program; activities are never run on a Friday which is “funeral day”, and it is accepted that people may often be away from the program to attend a funeral of a family member. Many Heart Health participants, however, were unable to travel back home to attend funerals due to health issues, and stated that one of their motivators of attending was to improve their health to be able to travel and to be able to properly grieve and pay their respects.

I haven’t been able to go back home for funerals and that because it’s a nine-hour drive and doctor wouldn’t let me travel. I ended up travelling to go to my sister’s funeral. But then I couldn’t go to my brother’s. It was too far for me at the time.

My Nanna passed away now. I can’t go to her funeral because it’s the same time as my Auntie’s.

For those with health issues, cultural obligations around funeral attendance can impact on appointment attendance and treatment compliance, and it is critical that health programs are sensitive to this as part of their cultural safety.

### 3.6. Kinship and Other Family Obligations

For Aboriginal people, “family” often extends beyond the traditional nuclear family, where great-aunts and great-uncles are considered grandparents, and nieces and nephews are considered children [54]. With the removal of thousands of Aboriginal children from their families in what became known as the Stolen Generation, many Aboriginal people in Australia have endured the legacy of severed family connections [35]. An unanticipated positive outcome of the Heart Health program has thus been the unplanned reunification with long-lost family members. This was spontaneously mentioned by many participants when asked about what they like about the Heart Health program, and several participants used the cameras to take photos of themselves with relatives that they had reconnected to through the Heart Health program. The inter-generational participation in the program was often commented on positively:

We have a lot of our oldies here in their nineties or nearly nineties and they’ve got their children there. So it is that family thing of keeping going.

I met a few other relatives at Heart Health. So I’m always bringing my family tree with me too now so we all sit down and talk about…

The strength of kinship ties is also advantageous to program recruitment, with word of mouth promotion by family members often mentioned as the conduit for initial program attendance:

…just telling other people about it; my cousins come for instance. I’ve got a whole lot of cousins from different sides of the family that come here…my mum started coming here before I did…I’m trying to get my sister to come at the moment.

The Heart Health program has worked hard to be responsive to the kinship ties and obligations of its participants. In the school holidays, for example, there are often activities that are tailored to family involvement, and throughout the year, it is not uncommon for a participant to come along with a child or grandchild “in tow”. This accommodating flexibility is intentional, as the program facilitators recognise that it is not always possible to predict when people will need to prioritise caring for family members. By enabling individuals to bring along family, it not only further reiterates the welcoming nature of the program, but it instils a sense surety in participants that they do not have to miss out on their Thursday sessions due to unexpected circumstances arising.

*What we do is just what every other program is doing around the place. We check everybody’s blood pressure, we check everybody’s sugars—but the how we do it is a bit different. The how is it’s culturally safe. You’re not right or wrong whether you come in for five minutes or two hours. If you want to walk in and out and rant and rave then that’s fine*.

## 4. Discussion

It has been noted that “even the best designed program will fail to achieve the desired outcomes if participants do not attend” [55]. This is particularly apt in the context of Aboriginal health, where non or low attendance in programs is a common lament [19,56]. As illustrated in this paper, however, exceptions lie in programs developed with and by Aboriginal people that are grounded in cultural safety, and sensitive to the social determinants of health. The Heart Health program has been operating weekly since its inception in 2009, with average attendance of 38 people per week (ranging from 29–50 people) [57], and many participants engaged as a result of the word of mouth testimony of family and friends.

This research and photovoice project began with the question, “what is it about the Heart Health program that sets it apart from other health promotion programs that seek to engage Aboriginal people?” In exploring this question with participants and program staff, two dominant and inter-related themes emerged. Firstly, and as examined in this paper, underlying social determinants not only shape health inequalities and health behaviours, but also draw attention to opportunities for more tailored intervention. In our view, the Heart Health program provides a pragmatic demonstration of strategies for embedding social determinants into what is ostensibly a health promotion and chronic disease prevention program. Moreover, the sustained participation and longevity of the program reinforces the way in which greater attention to social determinants can in-turn foster an environment that is more conducive to health education and health behaviour change. Secondly, the Heart Health program exemplifies the way in which “the small practical things” can make an enormous difference to program participation. Attendance flexibility, the creation of a physically “safe place”, and the imperative for cultural appropriateness in all activities are some examples of this identified through this research.

Culturally responsive and flexible healthcare delivery needs to be the basis of any health service training and implementation. This encourages health professionals to effectively engage with Aboriginal and Torres Strait Islander patients, including responding to differing perceptions of health, wellbeing, illness, and the body. There is a need for direct input from the community regarding program content enabling content to not only remain relevant to participants, but to also enable a sense of ownership over the program. Heart Health is unique in that it has been designed to meet the requirement of its participants via information gathering in yarning sessions, allowing individuals to raise health concerns and share their own experiences among peers. For many participants who have highly complex health issues, this is their most regular form of contact with healthcare professionals, hence Heart Health becomes an entry point to much broader health promotion and early intervention, as such, content is often reiterated and delivered in differing ways to reinforce core messages. While the primary aim of this evaluation was to explore barriers and enablers, regular attendance at Heart Health in itself is a risk prevention method. Through the provision of weekly monitoring of blood pressure and blood sugar levels, provision of healthy meals and encouraging participants to engage in physical activity, links to changes in health outcomes should be explored in future studies.

Further, many well intended health programs for Aboriginal people have failed because they do not give sufficient credence to the entrenched influence of such social determinants of health. As reflected in interviews with participants, the experiences of colonisation, discrimination, separation from family, and denial of education can impact adversely in a raft of ways. Social determinants play a substantial role in determining health and longevity [58]. Where we live, adverse childhood experiences, and access to education and employment are just some of the social factors that significantly impact on health [59]. Therefore, health service providers need an understanding of the historical and social context that allowed the disparities in social determinants to arise and engage with the community and together envisage a way forward.

### Limitations

The authors recognise that Aboriginal is a broad term used to describe numerous cultures with differing and unique traditions, histories, beliefs, and values. Whilst this research was undertaken predominantly with one cultural group in urban Western Australia, the significant impact of contextual social determinants on Indigenous health and wellbeing is mirrored in other countries, including Canada [31], New Zealand [60], and Brazil [61], and may therefore be applicable in other settings both nationally and internationally.

The authors also recognise that contextual social determinants of health are not limited to the six themes discussed in this paper, but merely demonstrate the importance of why they need to be considered in Aboriginal health promotion program planning. 

Due to the program design and lack of formal referral processes, it was not possible to capture the views of someone who was referred and did not participate in this program.

## 5. Conclusions

While the initial aim of this evaluation was to elicit the motivators, enablers, and barriers to Heart Health program participation, through yarning sessions, many participants made unprompted reference to the impacts of white settlement, discrimination, and the forced fracturing of Aboriginal families. These comments further cement the need for social determinants to be considered in the planning and implementation of health promotion programs.

Social determinants of health are extremely significant in the context of cardiovascular disease and other chronic diseases. As evident in the literature and among the participants in the Heart Health program, lack of education, normalisation of unhealthy behaviours, and entrenched trauma increase the prevalence of chronic disease risk factors such as unhealthy diet and risky use of tobacco, alcohol, and other drugs as coping mechanisms. These social determinants, in conjunction with a reluctance to trust authorities, also create barriers to accessing healthcare services for prevention, treatment, and rehabilitation. There are substantive learnings for other Aboriginal health promotion programs that can be gleaned from the insights of this program which was designed with, not for Aboriginal people.

Findings from this project offer greater insight into the benefits of developing culturally appropriate health programs and services, contribute to practice driven evidence in health promotion, and provide greater empowerment of Aboriginal people. Heart Health provides a safe flexible space that facilitates conversation, openness, and sharing.

## Figures and Tables

**Table 1:** Comparison of the Heart Health program and traditional hospital cardiac rehabilitation programs.

	Heart Health Program	Traditional Hospital Cardiac Programs
Referral	No formal process, mostly word of mouth or opportunistic due to location.	Formal referral from doctor or cardiac nurse post cardiac event.
Attendance	As often as wanted, program runs weekly. No specific attendance required.	Set period, usually 4–6 weeks long. Attendance at every session usually required.
Health prerequisites	Nil, program provides information on any health issues requested by participants with primary focus on cardiac issues and diabetes. Do not need to have any chronic diseases or cardiac issues to attend.	Post cardiac event.
Transport	Provided to anyone who requires it at no cost.	Make own arrangements.
Food	Morning tea and lunch provided.	Not provided.
Exercise	Group walk and exercise-physiologist led rehabilitation exercises (walk pending weather).	Dependent on program.
Structure	Diabetes round-table discussion, followed by a group walk and morning tea. Group yarning on weekly health topic (session can be facilitator led, participant led, or a more formal presentation style talk depending on topic and presenter); lunch is provided during this session. Followed by weight exercises. Finished with a cardiac-specific discussion; sharing of personal experiences and asking questions is encouraged. Participants are free to leave at any point. Blood pressure and insulin measured throughout the day.	Typically PowerPoint presentation while seated at desks.
